# Supplementary material for: Ag Nanoparticles Decorated CuO@RF Core-Shell Nanowires for High-Performance Surface-Enhanced Raman Spectroscopy Application
Source: Molecules. 2022 Dec 2;27(23):8460. doi: 10.3390/molecules27238460 (PMC9736506; doi:10.3390/molecules27238460)
Supplement: Supplementary file 1 [file molecules-27-08460-s001.zip › molecules-2002781-supplementary.pdf]

molecules

Supplemental Information

Ag Nanoparticles Decorated CuO@RF Core-Shell Nanowires for High-Performance Surface-Enhanced Raman Spectroscopy Application

Tung-Hao Chang [1,2,3], Hsin-Wei Di [4], Yu-Cheng Chang [4,*], and Chia-Man Chou [5,6,7,*]

[1] Department of Radiation Oncology, Changhua Christian Hospital, Changhua, 50006, Taiwan,
[2] Department of Radiological Technology, Yuanpei University, Hsinchu, 30015, Taiwan,
[3] Department of Medical Imaging and Radiological Sciences, Central Taiwan University of Science and Technology, Taichung, 40601, Taiwan,
[4] Department of Materials Science and Engineering, Feng Chia University, Taichung 407102, Taiwan;
[5] Department of Surgery, Taichung Veterans General Hospital, Taichung, 40705, Taiwan;
[6] College of Medicine, National Yang Ming Chiao Tung University, Taipei, 11221, Taiwan;
[7] Department of Post-Baccalaureate Medicine, National Chung Hsing University, Taichung, 40227, Taiwan
* Correspondence: yuchchang@fcu.edu.tw, cmchou@vghtc.gov.tw

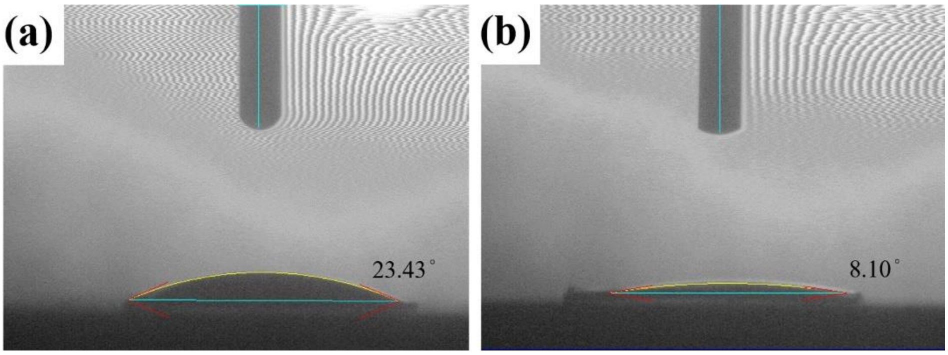

Figure S1 Contact angle images for water droplets on the surface of (a) CuO nanowires and (b) CuO nanowires with APTMS layer.

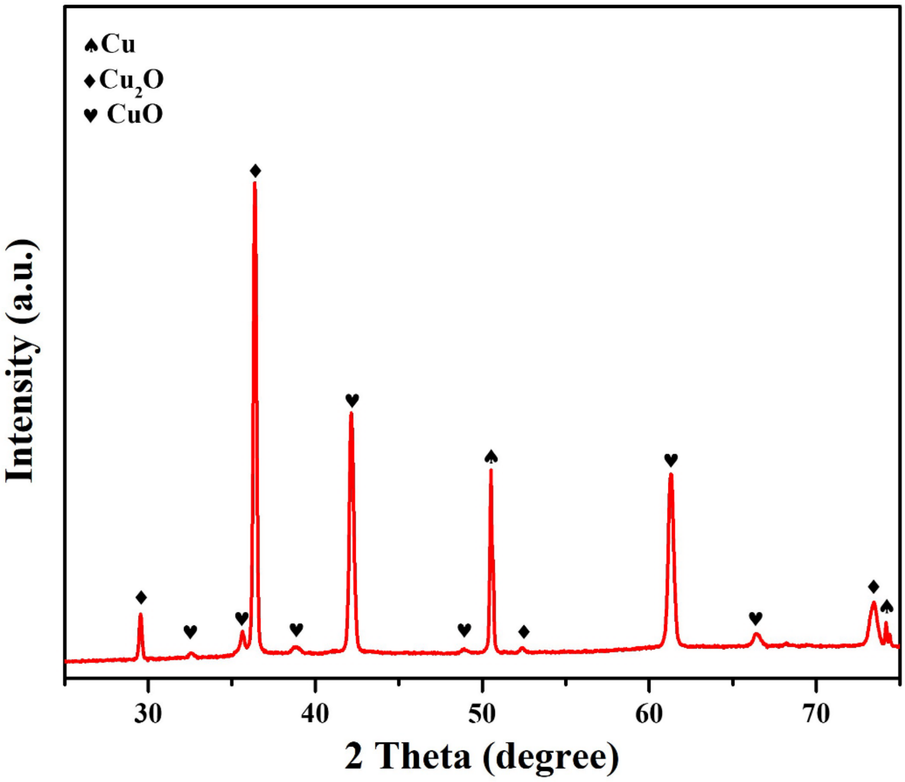

Figure S2 XRD diffraction pattern of CuO@RF@Ag nanowires grown at the resorcinol weight of 16.37 mg and $AgNO_3$ concentration of 0.45 mM.

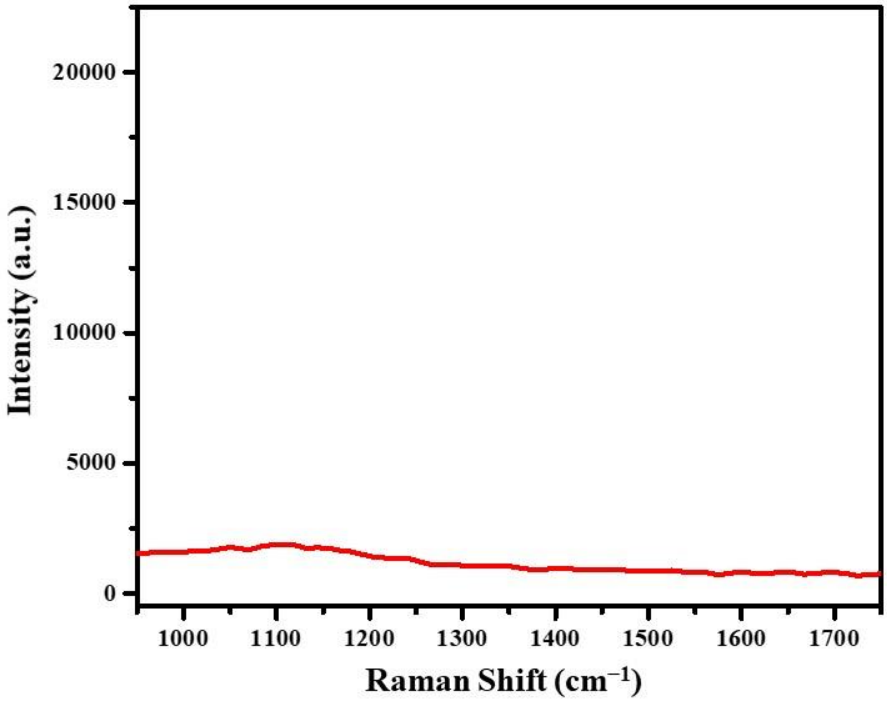

Figure S3 The Raman spectrum of CuO@RF nanowires.

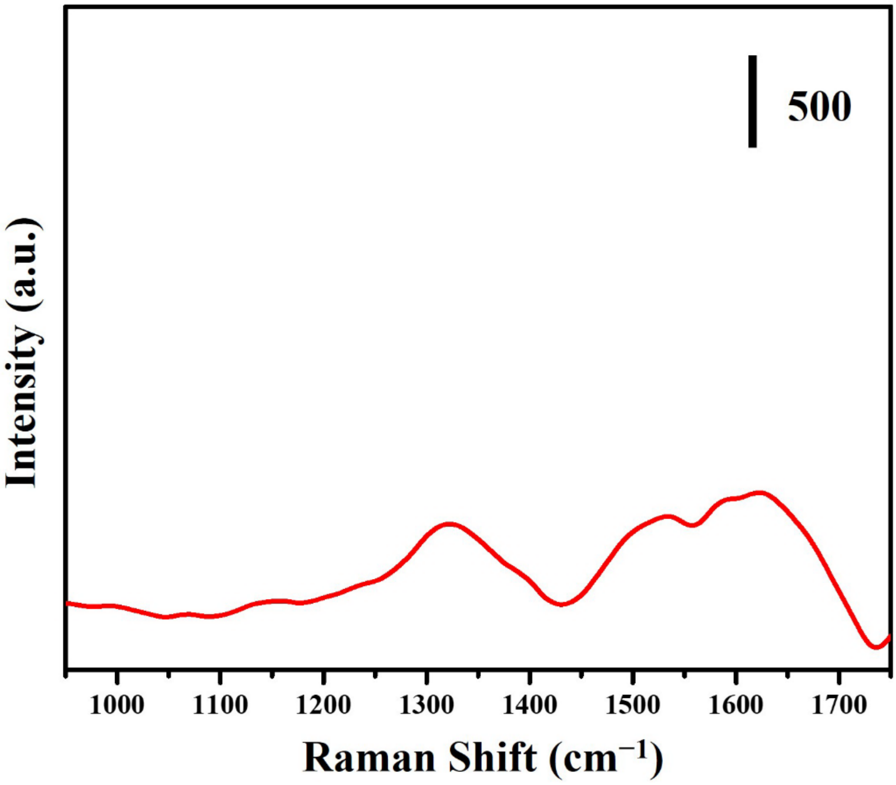

Figure S4 SERS spectrum of R6G at 10^{-13} M was obtained from CuO@RF@Ag nanowires.

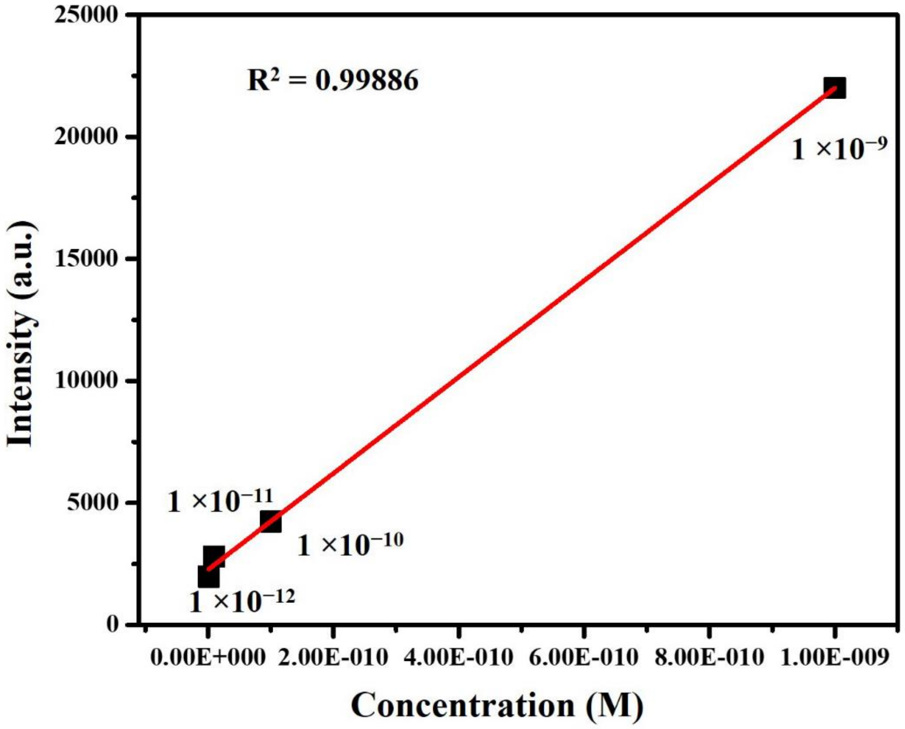

Figure S5 The concentration dependence of R6G peak intensity at 1649 cm^{-1} as a function of R6G concentrations ranging from 10^{-9} to 10^{-12} M for CuO@RF@Ag nanowires.

Table S1 Comparison of different methods for amoxicillin and 5-FU detection.

Materials	Drug	Detection Limit (M)	References
Ag nanoparticles	Amoxicillin	4.4×10^{-10}	Ref. 40
Ag nanocube	Amoxicillin	1×10^{-9}	Ref. 41
AuNPs/Si nano column	Amoxicillin	1×10^{-9}	Ref. 42
CuO@RF@Ag nanowires	Amoxicillin	1×10^{-10}	This work
Ag nanoparticles	5-fluorouracil	1×10^{-5}	Ref. 43
Ag colloid droplets	5-fluorouracil	3.8×10^{-7}	Ref. 44
Ag NPs/Si pyramids	5-fluorouracil	1×10^{-6}	Ref. 45
CuO@RF@Ag nanowires	5-fluorouracil	1×10^{-7}	This work
